# Honey bees increase their foraging performance and frequency of pollen trips through experience

**DOI:** 10.1038/s41598-019-42677-x

**Published:** 2019-05-01

**Authors:** Simon Klein, Cristian Pasquaretta, Xu Jiang He, Clint Perry, Eirik Søvik, Jean-Marc Devaud, Andrew B. Barron, Mathieu Lihoreau

**Affiliations:** 1 Research Center on Animal Cognition (CRCA), Center for Integrative Biology (CBI); CNRS, University Paul Sabatier, Toulouse, France; 2 Department of Biological Sciences, Macquarie University, NSW, Australia; 3 Honeybee Research Institute, Jiangxi Agricultural University, Nanchang, Jiangxi 330045 P.R. China; 4 Department of Biological and Experimental Psychology, School of Biological and Chemical Sciences, Queen Mary University of London, London, E1 4NS UK; 5 Volda University College, Department of Science and Mathematics, Volda, 6100 Norway

**Keywords:** Behavioural ecology, Animal behaviour, Entomology

## Abstract

Honey bee foragers must supply their colony with a balance of pollen and nectar to sustain optimal colony development. Inter-individual behavioural variability among foragers is observed in terms of activity levels and nectar *vs*. pollen collection, however the causes of such variation are still open questions. Here we explored the relationship between foraging activity and foraging performance in honey bees (*Apis mellifera*) by using an automated behaviour monitoring system to record mass on departing the hive, trip duration, presence of pollen on the hind legs and mass upon return to the hive, during the lifelong foraging career of individual bees. In our colonies, only a subset of foragers collected pollen, and no bee exclusively foraged for pollen. A minority of very active bees (19% of the foragers) performed 50% of the colony’s total foraging trips, contributing to both pollen and nectar collection. Foraging performance (amount and rate of food collection) depended on bees’ individual experience (amount of foraging trips completed). We argue that this reveals an important vulnerability for these social bees since environmental stressors that alter the activity and reduce the lifespan of foragers may prevent bees ever achieving maximal performance, thereby seriously compromising the effectiveness of the colony foraging force.

## Introduction

Social insects are reliant on the forager caste to supply resources for the whole colony^1^. However, not all foragers contribute equally to the collective effort of colony provisioning^2–4^, which raises the questions of how and why such inter-individual variability is observed.

In social bees, adequate colony nutrition requires a supply of both pollen (rich in proteins and fat) and nectar (simple carbohydrates)^5^. It has been argued that having different individuals specialised for nectar or pollen collection is the most efficient strategy at the colony level^6^, due to the different spatio-temporal distributions of these major nutritional resources in the field and to the need for specific behavioural skills to collect each of them. This is generally assumed to be true for honey bees where pollen and nectar foragers are considered as different behavioural castes^7–11^ that differ in their brain neuropeptide profiles^12^, sucrose response threshold^13^, ovary size^14^, levels of vitellogenin (a yolk precursor protein)^15^ and responses to social stimuli (pollen foragers rely more on dance communication)^16^. Together with evidence for genetic variation^8,17,18^, these observations suggest that pollen and nectar collection are evolved specialisations within the foraging force of the colony^17,19,20^. However, recent behavioural studies suggest that the distinction between pollen and nectar foraging may not be absolute. A proportion of the foragers seem to collect both resources, or may change specialisation as they age^21,22^. These studies, either conducted on a very limited number of individuals (less than 30 bees)^22^,or in spatially restricted flight cages with a few abundant artificial pollen or nectar sources^21^, call for further investigations. So far, no study has analysed the long-term foraging preferences of a large cohort of honey bees in the natural environment, and thus, data on how foragers partition their effort between pollen and nectar collection across their lifetime are limited.

In addition to variation in the type of resource collected by foragers^7–13^, some individuals forage more actively than others^21,23^. Tenczar *et al*.^21^ reported that the majority of foraging trips were performed by a minority of honey bee foragers, which they termed “elite bees”. In this study, however, the authors were not able to measure bees’ foraging performance in terms of amounts or rates of nectar or pollen collection, but only on the number of trips completed. Without this information, it is not possible to know if the most active foragers are also the most successful. One potential cause of inter-individual variation in foraging performance is the amount of experience gained by foragers over previous trips. Dukas^22^ and Schippers *et al*.^24^ both showed that individual honey bees improve their foraging performance with experience. While both studies recorded individual behaviour in detail across the whole foraging career of individuals bees, they did so on a rather limited sample size and focused on nectar resources only.

**Figure 1:**
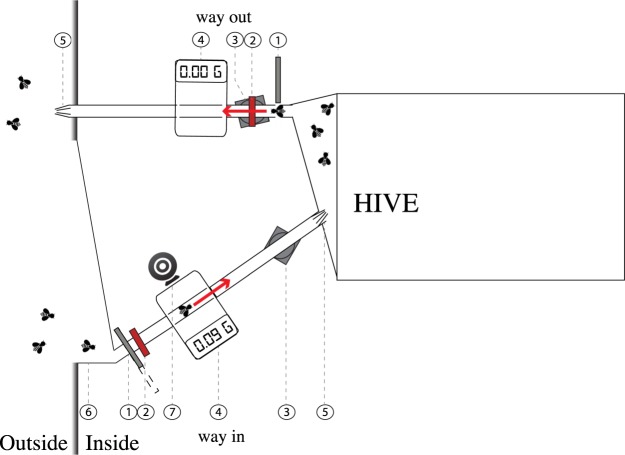
Colony entrance with sensors. Bees entered and departed the hive using two different paths. On each path, the bees were individually recognised (RFID), weighed (balance) and filmed (webcam). The entrance and exit tubes were 1 cm diameter transparent plastic tubes. (1) automatic gates, (2) infrared emitter/receiver, (3) RFID antennae, (4) balance, (5) Plastic bristles (forcing the passage of a bee from one direction only), (6) landing platform (open on the outside), (7) webcam.

Here, we explored the nature and possible causes of variation in foraging activity and performance between honey bee (*Apis mellifera*) foragers using an automated behavioural tracking system at the hive entrance to monitor the foraging behaviour of a large number of bees (>260) from two colonies in the natural environment (Fig. 1). The hives were located in a room and connected to the outside environment via a specially designed entrance containing baffles that forced bees to exit the hive along one path and to enter using a different path. Our system employed radio frequency identification (RFID) technology for the detection of arrival and departure times of individual bees at the colony entrance^25–31^ and interpolation of individuals’ foraging trips^25^. We also used a digital camera to photograph returning foragers and digital balances to record their mass. From these data, we analysed how individual bees differed in foraging performance throughout their entire foraging career.

## Results

### Bees varied in their tendency to collect pollen and non-pollen resources

We analysed the foraging activity of 564 bees for which we had information about the type of resource collected for at least one trip (Table 1). These bees performed an average of 19 foraging trips in their lifetime (mean ± SE, colony 1: 17 ± 1 trips, N = 295; colony 2: 21 ± 1 trips, N = 269) and their foraging span was less than a week (colony 1: 4.20 ± 0.18 days, N = 295; colony 2: 4.85 ± 0.18 days, N = 269).

**Table 1:** Number of foragers and foraging trips recorded per colony.

		Colony 1	Colony 2	All
Number of foragers	Non-pollen foragers	208	202	410
	Mixed foragers	87	67	154
	Total	295	269	564
Number of foraging trips	Non-pollen	2,159	1,076	3,235
	Pollen	371	138	509
	NA	2,399	4,380	6,779
	Total	4,929	5,594	10,523

The first five trips for each bee (orientation flights) were excluded. A bee was considered as ‘non-pollen forager’ if it had never collected pollen. A bee was considered ‘mixed forager’ if it had performed at least one trip for pollen. *NA* indicates trips with unknown identity of the collected resource (if any); bees recorded with only *NA* flights were excluded (see Material and Methods).

**Figure 2:**
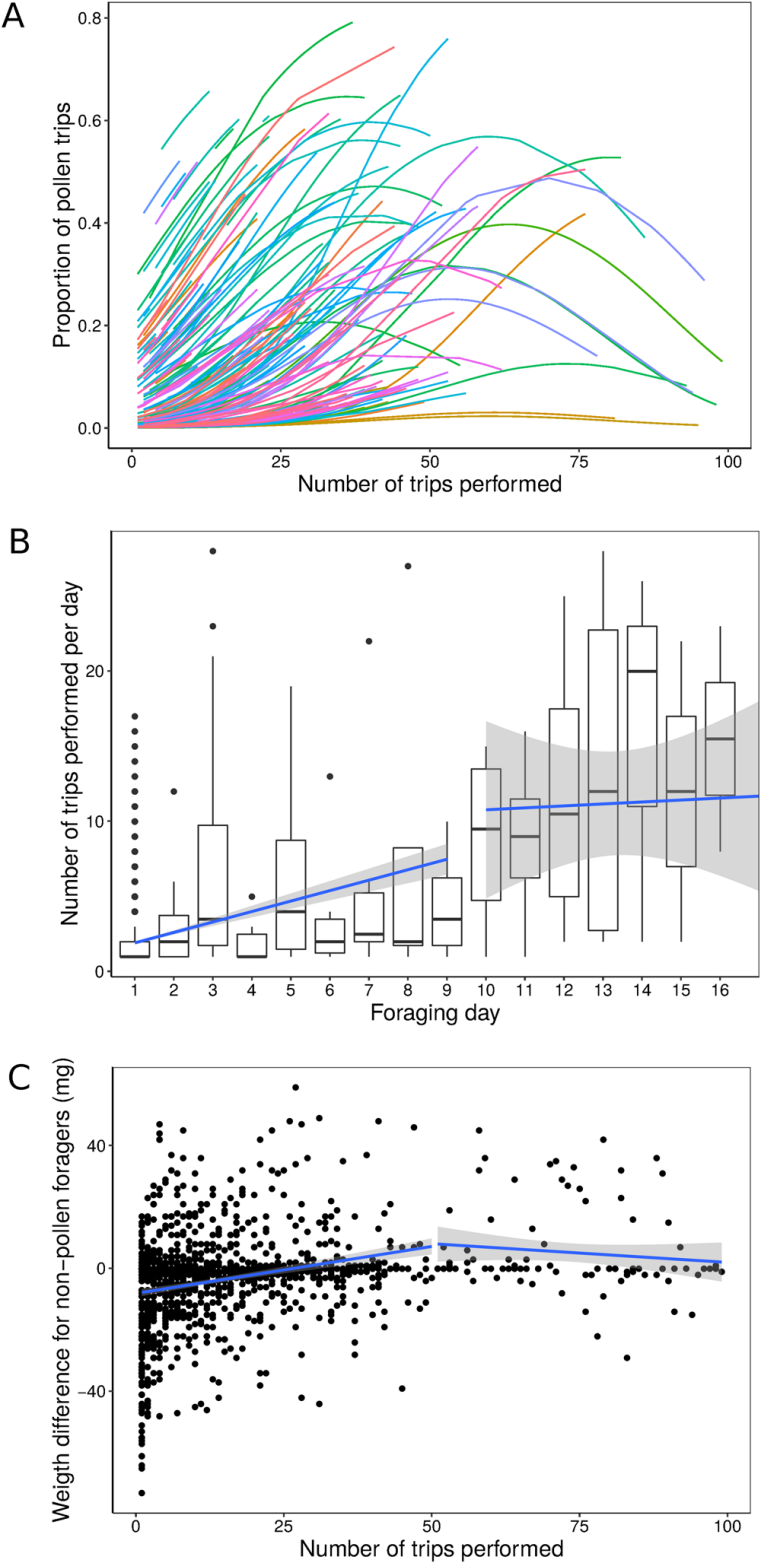
Changes of foraging performance with experience. (**A**) Probability for a bee to collect pollen on a given trip according to experience. Estimated curves for each bee according to a binomial GLMM are shown in colours (N = 154 bees, linear predictor Chi_1,2.215_ = 97.07, P < 0.001; quadratic predictor: Chi_1,2.215_ = 34.72, P < 0.001). (**B**) Total number of trips performed per bee across successive foraging days. Boxplot: the line shows the median; boxes and the whiskers represent interquartile ranges; dots represent outliers (data greater than third quartile + 1.5* (interquartile range), or less than first quartile − 1.5* (interquartile range)). Blue lines indicate the best fitted linear model obtained from a segmented regression analysis estimating break point at day number 9. (**C**) Foraging performance (weight difference between end and start of a non-pollen trip) per bee across successive trips. Bees performed better as they gained foraging experience. Only the non-pollen trips were analysed here (N = 277 bees). LMM: F_(1,925)_ = 6.45, P = 0.016.

On average 27% of bees foraged at least once for pollen (29% for colony 1, and 25% for colony 2, Table 1). None of these bees foraged exclusively on pollen. All of them performed a combination of pollen trips (cases when bees returned with pollen load) and non-pollen trips (cases when bees returned with no pollen load). We considered them as “mixed foragers”. Non-pollen trips could be unsuccessful trips, or trips for nectar or water. Incidences of pollen collection were distributed throughout the experiment and foragers varied in the number of trips as well as in the percentage of pollen trips they performed on different days (Fig. 2A).

For non-pollen trips the difference in mass on departure and return to the colony was either positive of around zero, and thus provides information about the amount of resources collected (mean ± SE, =−0.56 ± 0.24 mg, N = 925). For pollen trips the mean mass difference was negative (mean ± SE, −1.54 ± 12.5 mg, N = 136) despite bees returning with large and obvious pollen loads. From this, we infer that foragers returning with pollen must have expended mass (which we presume to be crop contents) during their pollen collection trip. Consequently, we could not use mass to measure the amount of pollen collected. Mixed foragers were heavier when leaving the hive for a pollen collecting trip than when leaving the hive for a non-pollen trip (Table 2b).

**Table 2:** Foraging strategies of mixed foragers.

	Estimate (SE)	df	t	P
**a**. *log*(*trip duration*) *~* *resource* + (*1* + *resource*|*day*) + (*1* + *resource*|*cohort*/*ID*)				
Intercept	**97**.**49** (**1**.**39**)	**7**.**67**	**38**.**20**	**<0**.**0001**
Pollen trips	**0**.**82** (**0**.**07**)	**81**.**75**	**−3**.**27**	**<0**.**0001**
**b**. *mass on departure* *~* *log*(*trip duration*) * *resource* + *colony* + (*1* + *log*(*trip duration*)|*day*) + (*1* + *log*(*trip duration*)|*cohort*/*ID*)				
NP trip intercept	**97**.**49** (**1**.**39**)	**59**.**90**	**70**.**00**	**<0**.**0001**
NP trip slope	**−1**.**22** (**0**.**37**)	**141**.**60**	**−3**.**27**	**0**.**001**
P trip intercept	92.01 (2.95)	952.50	−1.87	0.063
P trip slope	**1**.**44** (**0**.**95**)	**879**.**80**	**2**.**82**	**0**.**005**
Colony 2	**93**.**24** (**1**.**44**)	**68**.**20**	**−2**.**95**	**0**.**004**

(**a**) Summary of linear model representing the trip duration according to the type of resources collected by mixed foragers. Trip duration was natural-log transformed to obtain a Gaussian distribution. Significant effects are in bold. Model selection shown in Table S2A. (**b**) Summary of linear model representing the mass on departure according to the trip duration for mixed foragers. Trip duration has been natural log transformed to obtain a Gaussian distribution. Significant effects are in bold. Model selection shown in Table S2B.

### Bees improved their foraging performance and likelihood of pollen collection, with experience

For each bee, we found a quadratic relationship between the probability of collecting pollen on a foraging trip and the number of foraging trips already performed (LRTest: Chisq = 56.324, df = 1, P < 0.001). This indicates that bees tended to increase the collection of pollen during their first flights up to a maximum where the probability of pollen collection thereafter decreased with additional foraging experience (Fig. 2A, Wald ChiSq Test for Binomial GLMM; linear predictor Chisq_1,2.215_ = 97.07, P < 0.001; quadratic predictor: Chisq_1,2.215_ = 34.72, P < 0.001). The probability of collecting pollen was similar between the two colonies (Chisq_1,2.215_ = 3.03, P = 0.082). However, we found a stronger quadratic decline in the proportion of pollen collected with cumulative trips in colony 1 (tested during Australian autumn) than in colony 2 (tested in Australian spring) (Chisq_1,2.215_ = 11.84, P < 0.001), suggesting that environmental conditions influenced pollen availability and collection.

Foragers performed more trips per day until about day 9 after starting foraging (GLMM Poisson, Chisq_1,2.402_ = 9.32, P = 0.002). From this point, the number of trips performed each day per bee stabilised (GLMM Poisson, Chisq_1,23_ = 0.49, P = 0.484). Piecewise regression using the estimated break point at day 9 produced a better fit than a regression using the complete dataset (RSS_piecewise_ = 256661, RSS_complete_dataset_ = 280596, F = 60.99, P < 0.001, Fig. 2B)

Bees showed an increase in performance (weight difference between weight on arrival and weight on departure) for non-pollen trips with successive trip number (Fig. 2C, LMM, F_(1,925)_ = 6.45, P = 0.016). A similar relationship was observed for foraging efficiency (mass difference between departure and arrival divided by the trip duration, mg/min; Table S2, Fig. S2).

### Most foraging trips were performed by a minority of elite bees

**Figure 3:**
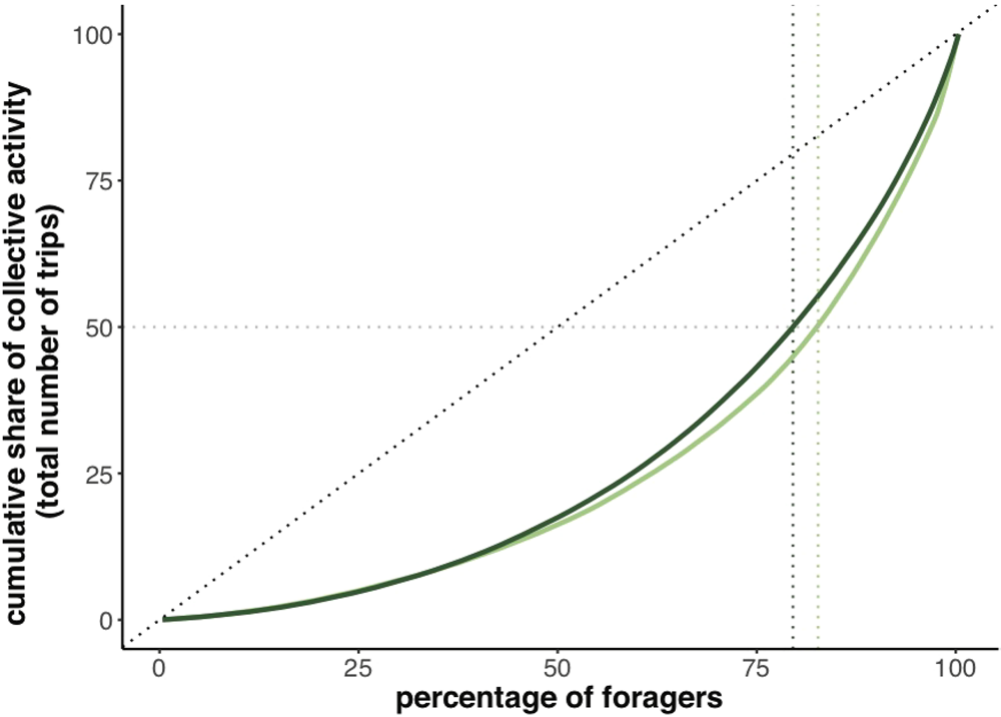
Lorenz curves of relative individual contributions to the colony foraging activity. For each colony, bees were ranked by the lifetime number of trips they performed in ascending order. The fraction of each bee’s contribution to the total number of the colony trips was cumulatively plotted in the Y axis. Black dotted lines represent the distribution predicted by an evenly distributed contribution of each bee. Grey dotted horizontal lines indicate the threshold of a contribution to 50% of the total activity. Vertical green dotted lines represent the fraction of foragers, for each colony, for which this threshold was reached. In colony 1, light green, (N = 296 foragers in total): 17.29% of the total of bees performed 50% of the total number of trips. In colony 2, dark green, (N = 270 foragers in total): 20.45% of the total of bees performed 50% of the total number of trips. See Fig. S3 for a similar analysis showing the proportion of foragers that contribute to the number of trips per bee per day.

We examined the proportion of foraging trips performed by each bee, relative to all the foraging trips of their colony. Visualisation of the inter-individual variation in foraging effort seen in each colony using a Lorenz curve^32^ indicates that around 19% of the tagged foragers performed 50% of the total number of trips recorded (17.29% for colony 1, 20.45% for colony 2; Fig. 3). Following Tenczar^21^, we describe these very active bees as elite bees. We then examined the skew of foraging activity between all foragers by computing a Gini index^33^ with values comprised between 0 (if all individuals contributed equally to the common task) and 1 (if only one individual performed the whole foraging task). We obtained a Gini index of 0.49 for colony 1 and 0.46 for colony 2, meaning that in both colonies, not all individuals contributed equally to the common foraging task.

### Elite bees performed better than non-elite bees

When comparing the performance of elite and non-elite bees we found a larger proportion (66%) of mixed foragers within the elite bees than within the non-elite bees (17.5%) (Fig. S1A, colony 1: 72% of elite bees are mixed foragers, *vs*. 20% of non-elite bees: of mixed foragers: χ^2^ = 55.68, df = 1, P < 0.0001; colony 2: 60% of elite bees are mixed foragers *vs*. 15% of non-elite bees: χ^2^ = 46.99, df = 1, P < 0.0001).

For non-pollen trips, elite bees had a greater gain in mass during their foraging trip than non-elite bees, indicating that elite bees returned more resources to the colony per trip (Fig. S1B). The differences in performance of elite and non-elite bees could be explained by the differences in experience between these two groups. Models considering individual experience on foraging performance showed a significant effect of experience, but no significant effect of elite bees as a fixed factor (Table S2).

## Discussion

We combined RFID tracking with video and mass recordings to explore the nature of variation in foraging performance between individual honey bees, and the possible causes of that variation. We found that foragers differed greatly in their lifetime foraging behaviour. Foraging performance and likelihood of collecting pollen increased as bees gained experience.

### Experienced foragers were more likely to collect pollen

In our study, scoring foragers that returned with pollen loads was unambiguous, but for bees that lacked pollen loads the nature of their trip was unclear. For bees that returned to the hive without pollen, we could not discriminate between an unsuccessful trip, a successful nectar collection or a successful water collection, and thus we classified trips as simply pollen or non-pollen. No bee collected pollen exclusively, which was concordant with other observations of behavioural introgression between pollen and nectar specialisations^13,21,22,34–37^. In our data, however, it was striking that pollen collection was performed by a minority of individuals, and usually only individuals that had accumulated substantial foraging experience. If these foragers are lost to the colony (due to predation, adverse weather or exposure to pesticides that disorient them^31^) there could be severe consequences for the pollen supply to the colony.

We note however that our experimental design was constrained by the use of colonies at different times of the year. The decline of pollen collection observed in experienced foragers of colony 1 (tested during Australian autumn) suggests that pollen availability was lower at this time of the year. In addition, we used small colonies with a minimum amount of brood because of the limitations imposed by the RFID sensors on forager traffic at the entrance. Future experiments on full-size commercial honey bee colonies at different periods of the year will be needed to assess the extent to which this pattern is dependent on season and population size.

### Elite bees undertook the majority of the foraging trips

All foragers tended to increase their number of foraging trips per day as they gained experience, until they reached a plateau of activity towards the end of their foraging career. However, all foragers did not contribute equally to the total foraging activity of their colony (Fig. 3). Rather we noticed a strong skew with a minority of very active bees (here and previously referred to as the elite bees^21^) undertaking the majority of the colony’s nectar and pollen foraging trips. In our study 19% of the foragers completed more than 50% of the total number of foraging trips in their colonies. In the same species, Tenczar *et al*.^21^ compared individual activity with total colony activity on a day-by-day basis because of a limit in the accuracy of their RFID sensor detection. When applying this particular analysis to our data (Fig. S3), we found 12% of the workers performed more than 50% of the colony’s daily activity, which is within the same range to the values reported by Tenczar *et al*. (around 20%)^21^.

### Elite bees performed better because they were more experienced

Prior social insect studies observing similar skews in foraging activity either failed to identify any particular link between performance and activity^21,23^, or simply reported a positive correlation between activity and efficiency^38,39^. In our data, we found a clear relationship between individual performance and experience (Figs 2 and S2). The simplest explanation for this is that through experience bees learn to improve their foraging performance, which is consistent with suggestions from earlier studies^22,24,40,41^. How this occurs is unknown, but cognitive studies have shown bees can learn to improve their foraging skills through trial-and-error^41^, their navigation between known food sources^42–47^, their discrimination of flowers (based on colours, shapes, odours or textures^48^) and their flower handling^49,50^. Any or all of these factors could explain the improvements in natural foraging performance with experience we observed in this study. Future experiments should also test whether environmental complexity (resource distribution, foraging distances, visual cues) affects the relationship between foraging experience and foraging performance, since individuals may learn more while making fewer trips in environments with complex structures.

### Implications for understanding the impact of stress on a colony

We showed that the most active and experienced bees are also the most efficient and high performing foragers in a colony. Tenczar *et al*.^21^ argued that elite bees can be rapidly replaced by a ‘reserve’ of less active foragers, but these authors did not examine individual foraging performance. Here we demonstrated that performance and efficiency is acquired through experience. Therefore, while the activity of an elite bee can be taken over rapidly by other foragers, these new foragers many not be as efficient until they have gained experience. Further, any stressors that shorten foragers’ lifespan, such as pesticides, pollutants, unbalanced diets, pathogens and viruses^31,51–61^, may prevent bees from accumulating enough experience to maximise their foraging contribution to the colony, ultimately leading to the recruitment of poorly efficient, precocious, foragers^62^. These interactions between stress, foraging force age composition, individual forager experience and performance deserve further study as they may help us understand the process of colony failure.

## Material and Methods

### Experimental hive

Honey bees (*Apis mellifera*) were obtained from the research apiary of Macquarie University (Sydney, NSW, Australia). The experimental hive was a four-frame nucleus hive (wooden box of 56 × 23 × 28 cm containing four standard Langstroth frames), placed in a dark room at the constant temperature of 24 °C. The hive contained two frames of honey and pollen, one frame of capped brood and one frame of polystyrene to fill the remaining space in the hive box.

The hive was connected to the outside environment via a specially designed entrance containing baffles that forced bees to exit the hive along one path and to enter using a different path (Fig. 1). Each path was made of transparent plastic tubes (1 cm diameter) that passed across an RFID antenna (Invengo, Guangzhou, China) and a microbalance pan (A&D Company Ltd, Japan). To prevent bees entering or exiting through the wrong tube, we attached inwardly tapering plastic bristles at the end of each tube. Bees landing at the entrance were funneled toward the single entrance tube.

Sections of the entry and exit tubes ran across dynamic micro balances of 1 mg sensitivity). The balances captured the mass of bees as they entered and exited the hive. Sometimes several bees crossed the balance at the same time and thus, the mass did not reflect the mass of just one individual. For this reason, we retained only values between 60 and 150 mg, which we evaluated as a conservative realistic individual mass range for honey bees. Examination of the videos of the entrance tunnel indicated that these limits were realistic.

Along the entrance path bees also passed beneath a webcam (Logitech), placed in a plastic box illuminated with white LED light, in order to video record the entrance tube. A motion detection video recording software (Netcam Studio X, Moonware Studios) was used to capture video footages of returning bees, thus allowing a visual assessment of the presence or absence of pollen in the corbiculae on the bees’ legs).

Automatic gates (micro-controlled servos connected to infrared emitter/receiver) regulated the traffic of bees within each path. The gates were placed at the beginning of the entrance and exit tubes. When a bee walked through the tubes and broke the beam of an infrared emitter/receiver, the connected gate would close behind the bee for 10 seconds. This time was an estimation of the maximum time needed for a bee to cross the RFID antenna and the balance. The infrared beams and gates were all connected and monitored using a single-board microcontroller system (Arduino, Adafruit Industries, USA).

### Experimental bees

This approach has been used to study bee behaviour for more than a decade^25^ to examine the impact of environmental stressors on bee foraging behaviour^26–31^, or to reveal individual foraging strategies^21,63^. RFID systems utilise tags, each with a unique digital ID that can be attached to bees. RFID tags were obtained from Invengo (Guangzhou, China). Each circular tag had a diameter of 4 mm and a mass of 1 mg and could be fixed to the bees’ dorsal thorax with glue (Loctite, Gel Super Glue). Each RFID tag had a unique 12-byte hexadecimal identifier that allowed us to track individual bees as they were detected by each antenna on exiting and entering the hive.

The hive was established with about 500 background (untagged) bees of mixed ages and a queen taken from a single colony. Newly emerged bees carrying individually programmed RFID tags were successively added to this hive. To source newly emerged bees we collected brood frames from up to eight different colonies over the course of the two experimental replicates to provide a diverse source of brood for the experiment. For each brood collection, frames were collected from two or three of the eight colonies. Brood frames were stored overnight in an incubator maintained at 37 °C. The next morning RFID tags were glued to the thorax of the newly emerged bees.

Tagged bees were added to the colony at different times. We defined a cohort as a group of bees tagged and added to the colony over up to four successive days. Each cohort came from a combination of different hives in order to increase diversity. Over the five weeks of the experiment, the hive received around 4,400 bees in total distributed within three cohorts. Approximately 500 tagged bees were added every day for the first four days of the experiment (first cohort). From day 21 of the experiment a further 1,800 tagged bees were added over four days (450 bees a day, second cohort). From day 35 of the experiment a further 600 bees were added over four days (150 bees a day, third cohort).

The experiment was conducted twice: colony 1, from April-May 2015 (Australian autumn); colony 2, from November-December 2015 (Australian spring). In colony 1, the queen died after two weeks and was replaced with a queen mandibular pheromone substitute (BeeBoost, Hornsby beekeeping supply, Australia).

Bees foraged in the surrounding suburban Australian environment including several nature reserves and private gardens. Such an environment provided nectar and pollen flow during the two seasons of the experiment, with a predominance of flowering native trees and bushes such as different eucalyptus species.

### Collating data on bee trips

RFID data for each trip included the RFID identification for the individual, the date and time it left the hive, and the date and time of its return to the hive, thus enabling us to calculate the duration of trips. Trips that lasted less than 10 s were considered as non-foraging trips^62^ and removed from the dataset. RFID readings were time-matched with readings from the balances to capture the mass of bees on departure and return to the colony. Difference in both mass measures gave an indication of resource collection (positive values) or expenditure (negative values). Videos taken up to 20 s before an entry RFID detection were inspected to score whether the tagged returning bee carried pollen (P) on its legs (see Supplementary Materials Video S1 in Dataset S2), no pollen (NP, see Supplementary Materials Video S2 in Dataset S2) or could not be reliably scored (NA) (i.e. when the bee legs were occluded on the video due to body angle or to the simultaneous presence of other bees in the entrance tube). The maximum time for a bee to travel from the webcam to the RFID antenna was visually assessed as 20 s.

### Data reliability

A total of 8,640 bees were tagged during the two replicates of the experiment (4,390 for colony 1 and 4,250 for colony 2). From the initial dataset, we excluded bees that performed only one trip and bees that had only ‘NA’ as load type over all their trips. In the final dataset, 3,432 bees (1,728 for colony 1 and 1,704 for colony 2) were retained. We speculate that the discrepancy between final bee counts and initial bees tagged is due to many bees losing their tags within the hive, some of the tags being damaged during the tagging process and rendered unreadable, and some bees not returning to the hive during their first trip, for reasons of health or inability to fly due to poor positioning of the tag. For each bee, we excluded the first five trips, which are more likely orientation flights than foraging flights^62^. A summary of our trip dataset is given in Table 1. The high number of ‘NA’ trips was mainly due to camera software issues, which was unfortunately most problematic for colony 2. The complete final data set is provided in Dataset S1.

### Data analyses

Data were analysed in R version 3.2.3 (operating via Rstudio, version 1.0.136^64^).

Differences between elite and non-elite foragers, in age at first foraging trip and in average mass on departure, were tested with a Wilcoxon rank sum test. Differences in the proportions of mixed foragers in elite and non-elite bees were tested with a Chi-square test.

The correlation between the average mass difference for non-pollen trips and bee activity was tested with a linear mixed model (LMM) using the function “lmer” in the package *lme4*^65^ (p-values were extracted using the package *lmerTest*^66^).

The correlation between the total mass differences for non-pollen trips and bee activity was tested using linear and quadratic mixed models, with “lmer”. We verified the existence of a quadratic relationship by comparing the linear and the quadratic models using a Loglikelihood Ratio Test (LRTest^67^). If the p-value was less than 0.05, we kept the model with the lower log-likelihood value.

Variation in mass difference for non-pollen trips according to the number of trips was tested using linear and logarithmic models with “lmer”. We verified the better fitting of the logarithmic relationship using the LRTest.

Variation in the number of foraging trips per day according to the number of foraging days were tested using a GLMM four count data with Poisson distribution error using the function “glmer” in *lme4*. After visual inspection the trend between the number of foraging trips as a function of foraging days revealed an apparent stabilization of foraging trips around after 9 days of experience. To test whether this stabilization was statistically significant, we fitted a piecewise mixed model splitting the dataset into two different regressions which break point was determined using the function “segmented” in the package *segmented*^68^. We compared the fits of the models (piecewise vs complete dataset GLMM) using the Residual Sum of Square (RSS) analysis. The lower the RSS the better the fit.

We estimated the probability of foraging for pollen according to experience (number of foraging trips) using a GLMM with Binomial distribution error, using the function “glmer”. We verified the existence of a quadratic relationship using the LRTest. Differences in mass at departure for non-pollen trips between non-pollen foragers (bees that did not perform any pollen trip) and mixed foragers (bees that collected pollen at least once) were analysed using a GLMM with Poisson distribution error.

For all models, colony identity was included as a covariate and bee identity nested in cohort identity was included as a random factor. Day identity was also included as a random factor to control for environmental variation between days. The colony of origin of the bees was found to have no effect when included as a fixed factor in the models. We thus did not include colony of origin as a potential explanatory variable in our analyses.

All minimum adequate models were selected by comparing their Akaike Information Criterion (AIC) to null models^69^ based on an analysis of variance (F test) in case of linear mixed model and analysis of deviance (Chisq test) in case of a generalized linear mixed model using respectively the function “anova” of the *stats* package and the function “Anova” of the *car* package (see details in Table S2). All the analyses of variance produced with the package *lmerTest* were run using the default Satterthwaite degree of freedom approximation^70^.

## Supplementary information

supplementary information

Supplementary Dataset 1
