# Supplementary material for: Honey bees increase their foraging performance and frequency of pollen trips through experience
Source: Sci Rep. 2019 May 1;9:6778. doi: 10.1038/s41598-019-42677-x (PMC6494865; doi:10.1038/s41598-019-42677-x)
Supplement: Supplementary file 1 — supplementary information [file 41598_2019_42677_MOESM1_ESM.pdf]

Honey bees increase their foraging performance and frequency of pollen trips through experience

Simon Klein[1,2], Cristian Pasquaretta[1], Xu Jiang He[3], Clint Perry[4], Eirik Søvik[5], Jean-Marc Devaud[1], Andrew B. Barron[2*] & Mathieu Lihoreau[1*]

[1] Research Center on Animal Cognition (CRCA), Center for Integrative Biology (CBI); CNRS, University Paul Sabatier, Toulouse, France

[2] Department of Biological Sciences, Macquarie University, NSW, Australia

[3] Honeybee Research Institute, Jiangxi Agricultural University, Nanchang, Jiangxi, P.R. of China, 330045

[4] Department of Biological and Experimental Psychology, School of Biological and Chemical Sciences, Queen Mary University of London, London E1 4NS, UK.

[5] Volda University College, Department of Science and Mathematics, Volda 6100, Norway

[*] These authors contributed equally to the work

Corresponding author: andrew.barron@mq.edu.au

SUPPLEMENTARY MATERIALS:

This file contains the following supplementary information:

Figure S1: Comparative foraging efficiency of elite and other foraging bees

Figure S2: Foraging efficiency for non-pollen trips according to foraging experience

Figure S3: Lorenz curves of relative individual contributions to the colony foraging activity.

Table S1: Analyses of mass on departure.

Table S2: Selection of the best general linear mixed models based on their AIC.

Dataset S1 index: Raw data of each trip for every bee.

A

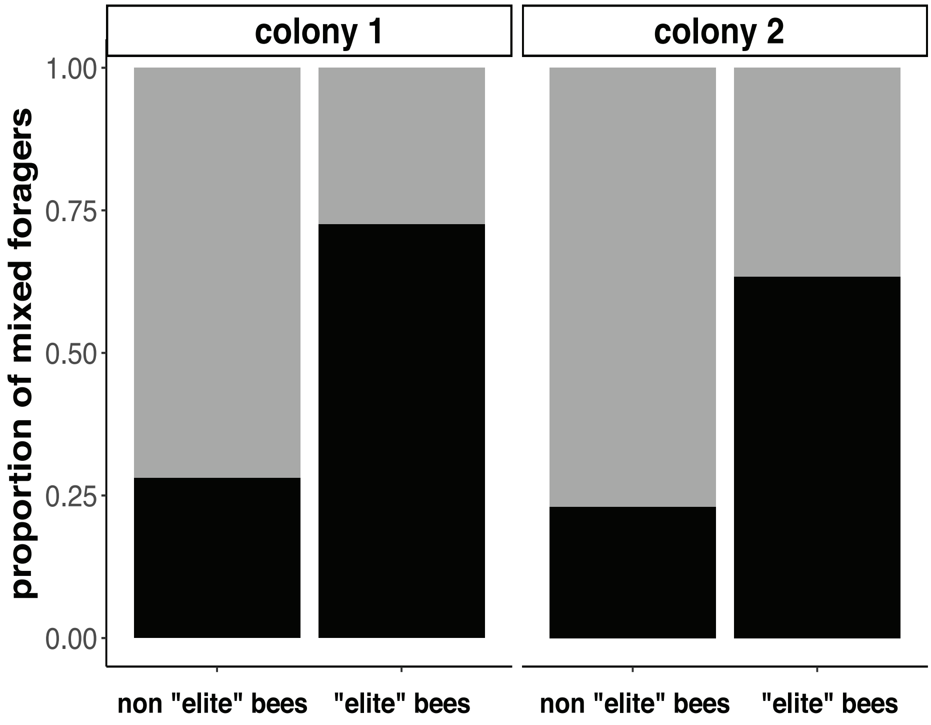

B

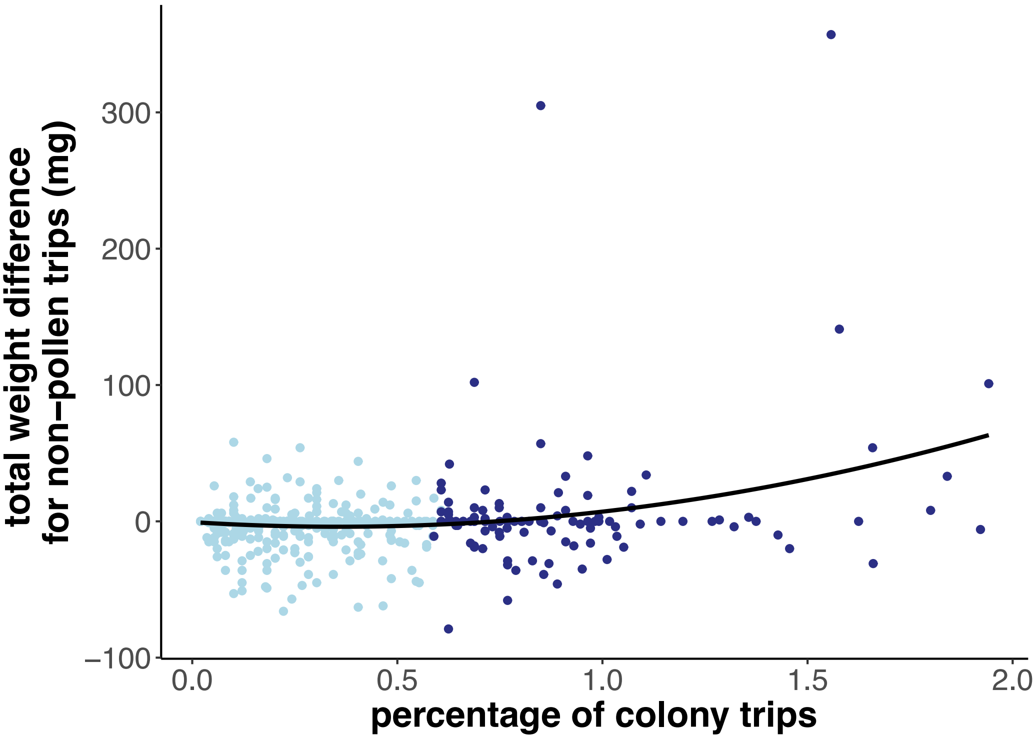

Figure S1: Comparative foraging efficiency of elite (*dark blue*) and other foraging bees (*light blue*) (N = 527 individuals, colony 1: 281, colony 2: 246)**. A**. Relative proportions of mixed foragers among elite bees and other foragers. While

non-pollen foragers (in grey) represent the majority of non-elite bees, elite bees are mostly mixed foragers (i.e. those that performed at least one trip collecting pollen, in black). (Left: colony 1: $\chi^2 = 55.68$, DF = 1, P < .0001; right: colony 2: $\chi^2 = 46.993$, DF = 1, P < .0001). **B.** Cumulated individual mass differences. The most active bees accumulated larger amounts of food. General quadratic model: $y \sim ax^2+bx+c$: a = 25.34 ± 5.54, DF = 20.16, T = 4.57, P < .0001; b = -19.27 ± 10.55, DF = 1887, T = -1.85, P = 0.068; c = 1.05 ± 15.69, DF = 835, T = 0.067, P = 0.947, elite: 1.85 ± 4.81, DF = 2062, T = 0.38, P = 0.699. Model selection in Table S2.

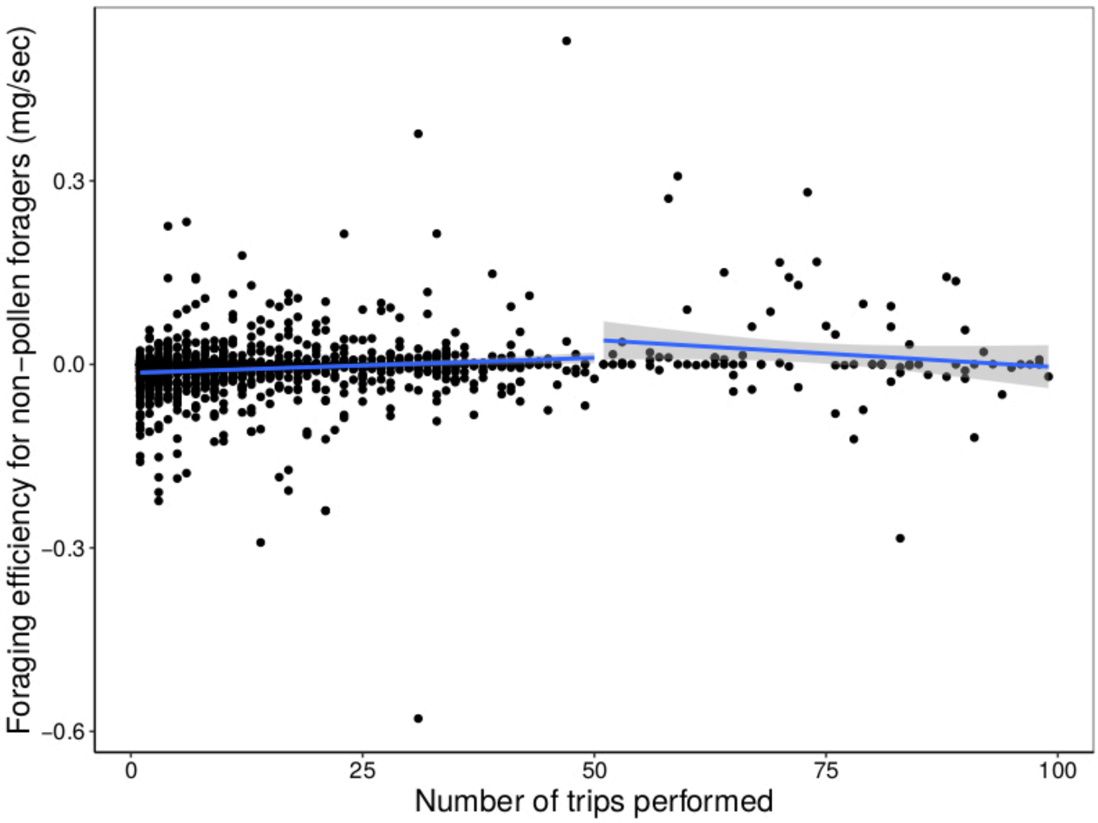

Figure S2: Foraging efficiency for non-pollen trips (mass difference between departure and arrival divided by the trip duration, mg/min). Piecewise regression analysis shows that bees seem to increase their foraging efficiency with experience until around trip number 50 (LMM, $F_{1,1221} = 12.99$, $P < 0.001$). Their foraging efficiency is stable thereafter (LMM, $F_{1,91} = 1.42$, $P = 0.237$).
Model selection in Table S2.

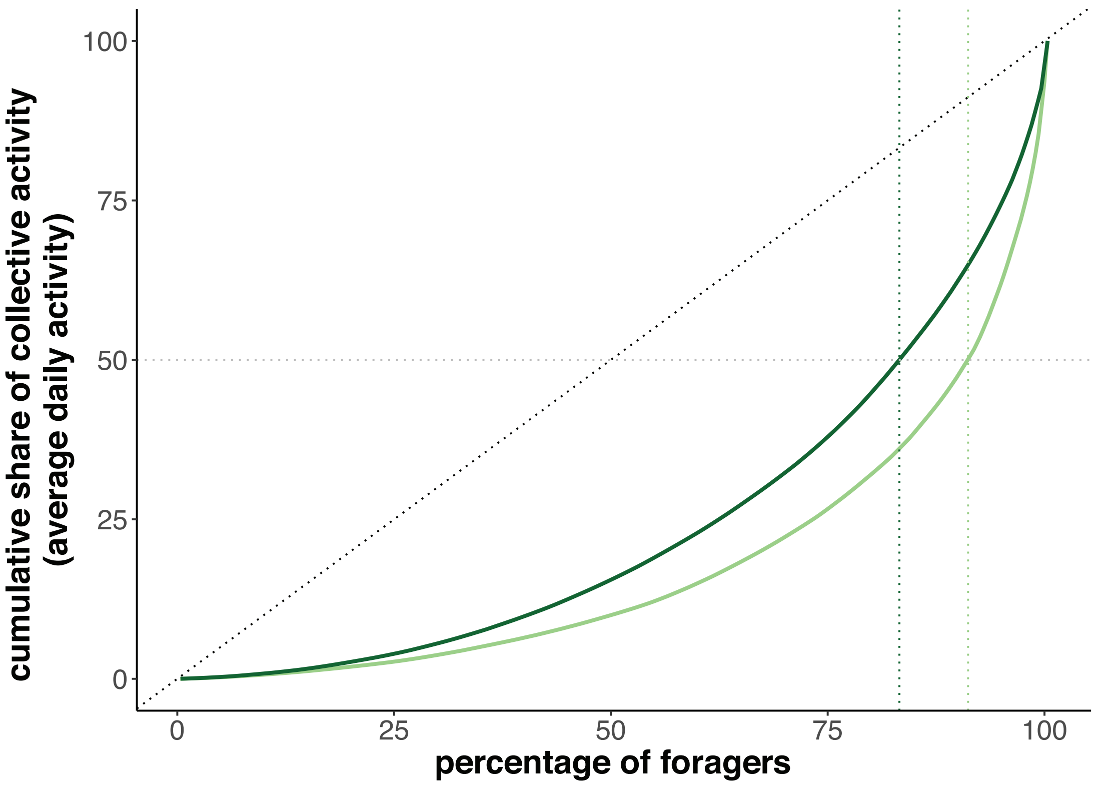

Figure S3: Lorenz curves of relative individual contributions to the colony foraging activity. Such curves present, for each colony, the proportion of foragers that contribute to the number of trips per bee per day compared to the total number of trips performed by the colony that day. For each colony (light green: colony 1, dark green: colony 2), bees were ranked by the relative daily activity they performed in ascending order, and the fraction of each bee's contribution to the total foraging activity was cumulatively plotted in the Y axis. Black dotted lines represent the distribution predicted by an evenly distributed contribution of each individual. Grey dotted horizontal lines indicate the threshold of a contribution to 50% of the total activity. Vertical green dotted lines represent the fraction of foragers, for each colony, for which this threshold was reached. Here, 8.81% (colony 1) and 16.73% (colony 2) of the foragers contributed to the total colony foraging activity

Table S1: Analyses of mass on departure. a. Summary of linear model looking at the mass difference upon departure for non-pollen trips, between non-pollen foragers and mixed foragers. There is no significant difference between the two groups (model selection in Table S2). **b.** Summary of linear model looking at the mass difference on departure for non-pollen trips and pollen trips, for mixed foragers (model selection in Table S2).

	**Estimate (SE)**	**df**	**t**	**P**
**a.** *mass on departure ~ forager type * colony + (1+forager type|day)+ (1+forager type |cohort/ ID)*				
**Non pollen foragers colony 1**	**94.56 (1.77)**	**3.36**	**53.3**	**<.0001**
Mixed foragers colony 1	95.01 (1.53)	2.06	0.31	0.786
Non pollen colony 2	93.31 (2.77)	3.41	-0.31	0.777
Mixed foragers colony 2	92.02 (2.53)	2.98	-1.00	0.390
**b.** *mass on departure ~ resource * colony + (1+resource |cohort / ID)*				
**Intercept colony 1**	**93.86 (0.84)**	**6.70**	**111.2**	**<.0001**
Resource colony 1	98.99 (2.72)	1.84	1.85	0.215
Intercept colony 2	91.27 (1.50)	17.55	-1.72	0.102
Resource colony 2	90.83 (4.29)	197.02	-1.86	0.186

Table S2: Selection of the best general linear mixed models based on their AIC. Best model is highlighted in bold.

	df	AIC	Loglik	Chi^2	P
**a.** Log(trip time) ~ resource	3	17592	-8793		
**Log(trip time) ~ resource + (1+resource)|day + (1+resource)|cohort/ID**	**12**	**5245**	**-2610**	**12364**	**<.0001**
Log(trip time) ~ resource + colony + (1+resource)|day + (1+resource)|cohort/ID	13	5245	-2609	2.1	0.15
Log(trip time) ~ resource * colony + (1+resource)|day + (1+resource)|cohort/ID	14	5247	-2609	0.1	0.746
**b.** Mass on departure ~ log(trip duration) * resource	3	7495	-3744		
Mass on departure ~ log(trip duration) * resource + (1+log(trip duration)) | day identity + (1+log(trip duration)) | cohort/ID	9	7334	-3657	173.6	<.0001
**Mass on departure ~ log(trip duration) * resource + colony + (1+log(trip duration)) | day identity + (1+log(trip duration)) | cohort/ID**	**11**	**7330**	**-3654**	**7.6**	**0.022**
Mass on departure ~ log(trip duration) * resource * colony + (1+log(trip duration)) | day identity + (1+log(trip duration)) | cohort/ID	14	7398	-3651	6.3	0.097

	df	AIC	Loglik	Chi^2	P
**c.** Total non-pollen income ~ percentage of colony trips + elite	3	5327.1	-2660.5		
Total non-pollen income ~ percentage of colony trips + elite + (1+ percentage of colony trips)|cohort/ID	5	5165.1	-2577.6	198.3	**<.0001**
**Total non-pollen income ~ exp(percentage of colony trips) + elite + (1+ exp(percentage of colony trips))|cohort/ID**	**5**	**5153.4**	-2560.6	**34.5**	**<.0001**
**d.** Intertrips duration ~ percentage of colony trips + elite	3	5049.7	-2521.8		
Intertrips duration ~ percentage of colony trips + elite + (1+ percentage of colony trips)|cohort/ID	5	4873.7	-2431.9	189.3	**<.0001**
**Intertrips duration ~ log(percentage of colony trips) + elite + (1+ log(percentage of colony trips))|cohort/ID**	**5**	**4853.6**	**-2421.8**	**20.13**	**<.0001**
*mass on departure ~ resource * colony + (1+resource |day) + (1+resource |cohort / ID)*					
**e.** Mass on departure ~ forager type	3	12530	-6204		
Mass on departure ~ forager type + (1+forager type|cohort/ID)	9	12338	-6135	132.2	<0.0001

	df	AIC	Loglik	Chi^2	P
Mass on departure ~ forager type + colony + (1+forager type|cohort/ID)	10	12290	-6135	1.9	0.169
Mass on departure ~ forager type * colony + (1+forager type|cohort/ID)	11	12291	-6135	1	0.319
**Mass on departure ~ forager type * colony + (1+forager type|Day) + (1+forager type)|cohort/ID**	**14**	**12284**	**-6128**	**13.4**	**0.004**
**f.** Mass on departure ~ ressource	3	-6057.8	3031.9		
Mass on departure ~ resource + (1+ resource |cohort/ID)	9	-6219.4	3118.7	173.60	**<0.005**
Mass on departure ~ resource + colony + (1+resource|cohort/ID)	10	-6220.4	3120.2	3.05	**0.081**
**Mass on departure ~ resource * colony + (1+resource|cohort/ID)**	**11**	**-6223.0**	**3122.5**	**4.60**	**0.032**
Mass on departure ~ resource * colony + (1+resource|Day) + (1+resource)|cohort/ID	14	-6223.3	3125.7	6.32	0.097
**g.** Resource ~ **colony** + (1+experience)|cohort/ID + (1|Day)	9	2758.3	-1370.2		
Resource ~ **(experience + experience^2)** + (1+experience)|cohort/ID + (1|Day)	10	2691.7	-1335.7	68.60	<.0001

	df	AIC	Loglik	Chi^2	P
Resource ~ **(experience + experience^2)** + colony + (1+experience)|cohort/ID **+ (1|Day)**	11	2692.1	-1335.0	1.66	0.198
**Resource ~ (experience + experience^2) * colony + (1+experience)|cohort/ID + (1|Day)**	**13**	**2679._**	**-1326.9**	**16.25**	**< 0.001**
**h.** Trip per day ~ day foraging	3	13798	-6896.3		
**Trip per day ~ day foraging + (1+day foraging)|day identity + (1+day foraging)|cohort/ID**	**12**	**13366**	**-6670.9**	**119.6**	**<.0001**
Trip per day ~ day foraging + colony + (1+day foraging)|day identity + (1+day foraging)|cohort/ID	13	13365	-6669.5	2.82	0.092
**i.** Mass difference ~ experience	5	7414.0	-3702.0		
**Mass difference ~ experience + (1+experince)|cohort/ID**	**9**	**7403.0**	**-3692.5**	**14.54**	**<.0001**
Mass difference ~ experience + colony + (1+experience)|cohort/ID	10	7405.0	-3692.5	0.03	0.862
Mass difference ~ experience * colony + (1+experience)|cohort/ID	11	7406.9	-3692.5	0.03	0.856

Dataset S1: Raw data of each trip for every bee.

Variables:

ID: identity of the bee based on its RFID tag number.
t_in: time and day of the bee entering the hive
t_out: time and day of the bee leaving the hive
day_out: day of the bee leaving the hive
day_in: day of the bee entering the hive
trip_time: trip duration (min)
w_out: mass on departure (g)
w_in: mass on return (g)
w_diff: mass difference for the trip (g)
colony: colony origin
cumul_trip: experience (number of trips performed)
pollen: resource, either pollen ‘p’, non-pollen ‘n’, or unknown ‘na’.
